# Correction: Hematological changes in the blood of experimental male and female albino rats on exposure to pesticide, dimethoate

**DOI:** 10.1371/journal.pone.0334064

**Published:** 2025-10-10

**Authors:** The PLOS One Staff

## Notice of Republication

This article was republished on September 16, 2025, to correct an errors in affiliation of the fourth author. The affiliation should read: Zoology Department, College of Science, King Saud University, Riyadh, Saudi Arabia

The publisher apologizes for the errors. Please download this article again to view the correct version. The originally published, uncorrected article and the republished, corrected articles are provided here for reference.

## Supporting information

S1 File Originally published, uncorrected article. (PDF)

S2 File Republished, corrected article. (PDF)
