# Supplementary material for: Hematological changes in the blood of experimental male and female albino rats on exposure to pesticide, dimethoate
Source: PLoS One. 2025 May 6;20(5):e0321848. doi: 10.1371/journal.pone.0321848 (PMC12083873; doi:10.1371/journal.pone.0321848)
Supplement: Attachment — Submitted filename: Reviewers decission.docx [file pone.0321848.s001.docx]

**Reviewer 1**

Concern over statistical analysis

authors have not made all data underlying the findings in their manuscript fully available

Study was performed to check effect of pesticide, dimethoate exposure on albino (Albino Rats) hematological parameters.

Study design has some defects, as it is not clear how much dose was administered to rats. Dosage mentioned in mL has very little understanding. Many of the references mentioned in the discussion do mention the dose in form per kg animals.
Lot of studies are mentioned in the discussion tells the effect of dimethoate pesticide on hematological parameters. There is no novelty in the study as MT Akay et al in 1999 mentioned similar observations (PMID: 10509431) All the speculations based on data from semi auto hematology analyzer. If these observations would have been supported with certain biochemical tests it would have great support to the data.
Research group has used both male and female rats for the treatment. However, in results it has not shown comparison of responses from male and female rats. Results are analyzed in very narrow spectrum of treated Vs non-treated.
There is lot of mismatches in description, table and figure

**Reviewer 2: Minor revision**

Nida Saleem et al. have shown in this article that, following exposure to the dimethoate pesticide for 30, 60, and 90 days in both male and female albino rats, the number of white blood cells, platelet count, granulocyte count, and lymphocyte count significantly increased. In contrast, the number of red blood cells, hemoglobin level, mean corpuscular hemoglobin (MCH), and mean corpuscular hemoglobin concentration (MCHC) were notably decreased. No changes were observed in the control group of male and female albino rats. This study concludes that dimethoate pesticide affects blood parameters in both male and female albino rats.
The article looks interesting to me. I have a few minor comments that could be incorporated to improve it.
1. Since Figure 3.1 appears before Figure 2, please correct the figure numbering so that the figures appear in proper sequence.
2. In Figures 3.1, 2, and 3 (all bar diagrams), label the Y-axis clearly to indicate what is being measured.
3. In the graphical abstract, please include the name of the dose for clarity.
