# Supplementary material for: Hematological changes in the blood of experimental male and female albino rats on exposure to pesticide, dimethoate
Source: PLoS One. 2025 May 6;20(5):e0321848. doi: 10.1371/journal.pone.0321848 (PMC12083873; doi:10.1371/journal.pone.0321848)
Supplement: Attachment — Submitted filename: Response to Reviewers.docx [file pone.0321848.s002.docx]

**Response to Editor**

Dear Editor,

Thank you for the opportunity to revise our manuscript in response to the reviewers' comments. We have carefully considered their feedback and made the necessary revisions to improve the quality of our manuscript. In particular, we have addressed the concerns raised about the clarity and organization of our work. The manuscript has been reorganized for better flow and coherence, and we have modified language and phrasing to enhance clarity.

We believe these revisions have significantly improved the manuscript, and we hope that both you and the reviewers find them satisfactory. We truly appreciate the insightful feedback and constructive suggestions that have been provided. The following is a detailed, item-by-item response to each of the reviewers’ points. Our replies are marked in yellow, and changes in the manuscript are indicated with added sentences highlighted or strikethrough for deleted sentences.

Additionally, we acknowledge your concerns regarding the quality of the English language used in the manuscript. We apologize for any confusion or inconvenience that may have occurred. We have taken your comments seriously and made extensive edits to address grammatical errors, improve sentence structure, and ensure consistency throughout the manuscript. We are confident that the revised version now meets the necessary language quality standards.

We hope the revised manuscript meets your expectations and adequately addresses all of your concerns. We would be grateful if you could review the manuscript again and provide us with any additional feedback you may have.

Once again, we would like to express our gratitude for your valuable feedback and guidance throughout the review process.

Sincerely yours,

**Response to Reviewers**

**Reviewer 1:**

**Concern over statistical analysis**

**Response:** We appreciate the concern regarding the statistical analysis used in this study. A one-way ANOVA was selected for dose comparison because it is a robust statistical method for analyzing differences among the means of four independent groups. In our study, the doses of dimethoate represent independent groups, and one-way ANOVA allowed us to determine whether there were statistically significant differences in the measured parameters across these groups.

Key justifications for using one-way ANOVA in this study are as follows:

Study Design: The experiment involved independent groups exposed to different doses of the substance, with each group being treated as a separate, independent category.

Comparison of Means: One-way ANOVA was specifically chosen to identify significant differences between the dose groups.

This approach ensures the reliability and accuracy of the statistical analysis, allowing for valid conclusions regarding dose-dependent effects. We believe this method appropriately addresses the objectives of our study and aligns with established statistical practices in similar research.

If further clarification or additional analyses are required, such as testing assumptions or using alternative statistical methods (e.g., non-parametric tests), we are willing to provide these upon request.

**Authors have not made all data underlying the findings in their manuscript fully available**

**Study was performed to check effect of pesticide, dimethoate exposure on albino (Albino Rats) hematological parameters.**

**Response:** We acknowledge the journal's concern regarding the availability of data underlying our findings. In this study, we assessed the effects of pesticide exposure (dimethoate) on the hematological parameters of albino rats. Due to ethical considerations, privacy concerns, we are unable to make the raw data fully available for public access at this time. However, we assure you that the data supporting our conclusions are included in the manuscript files.

We are committed to ensuring transparency in our research and are happy to provide the data upon reasonable request from the journal or any other interested researchers, with due consideration for confidentiality and ethical guidelines.

Should the journal require any additional clarification or specific datasets to be shared directly, we are open to discussing further solutions.

Thank you for your understanding.

**Study design has some defects, as it is not clear how much dose was administered to rats. Dosage mentioned in mL has very little understanding. Many of the references mentioned in the discussion do mention the dose in form per kg animals.**

**Response:** Thank you for pointing out the concern regarding the dosage administered to the rats in our study. We understand that specifying the dose in mL without a clear reference to body weight may lead to confusion. To clarify:

In our study, the dosages of dimethoate were administered based on the body weight of the rats. The amount of dimethoate given was carefully calculated to ensure appropriate dosing for each group. The doses were converted into concentration (mL/kg body weight) for accurate comparison with relevant studies.

For each treatment group, the doses were as follows:

Different concentrations of pesticides were prepared according to calculated LD_50_ (acute oral LD_50_ 0.085 ml). T1 group was treated with low concentration of pesticide ( of LD_50_), T2 group was treated with medium concentration of pesticide ( of LD_50_), T3 group was treated with high concentration of pesticide ( of LD_50_).

We apologize for the lack of clarity in the initial manuscript, and we have updated the manuscript to include these doses in mL/kg body weight to ensure better understanding. We have also included this clarification in the revised Methods section for further clarity.

We hope this addresses your concern, and we appreciate your valuable feedback. If further adjustments or additional details are needed, please let us know.

**Lot of studies are mentioned in the discussion tells the effect of dimethoate pesticide on hematological parameters. There is no novelty in the study as MT Akay et al in 1999 mentioned similar observations (PMID: 10509431) All the speculations based on data from semi auto hematology analyzer. If these observations would have been supported with certain biochemical tests it would have great support to the data.**

**Response:** We appreciate the journal’s valuable feedback and understand the concern regarding the novelty of our study in relation to previous research, particularly the work by MT Akay et al. (1999). While it is true that similar observations regarding the effects of dimethoate on hematological parameters have been made in earlier studies, we believe that our study offers new insights in several important ways:

1. **Extended Study Duration**: Unlike many previous studies, our study was conducted over a 90-day period, allowing us to observe the long-term effects of dimethoate exposure, which is often not fully explored in other studies.
2. **Sampling Frequency:**

In other studies, the duration is shorter, and sampling was done only once, whereas in my study, sampling was conducted every 30 days, and tests were performed accordingly. In this way, a total of 3 tests were conducted after 30, 60, and 90 days.

3. **Use of Autohematology Analyzer in the Study:**

In my study, an autohematology analyzer was used after manual calculations, whereas in other studies, a semi-auto hematology analyzer was used.

4. **Gender-Specific Analysis**: Our study uniquely compares the effects of dimethoate on hematological parameters between male and female albino rats, providing insights into potential gender-based differences in response to pesticide exposure.
5. **Contribution to Pesticide Regulation**: By highlighting the long-term, sub-lethal effects of dimethoate exposure, particularly with regard to gender-based differences in hematological parameters, our study provides critical data that could inform pesticide regulation and public health guidelines.

Regarding the suggestion to include biochemical tests, we recognize that biochemical support could strengthen our findings. While the primary focus of this study was on hematological parameters, we acknowledge that adding biochemical assays (e.g., liver enzymes, oxidative stress markers) would provide a more comprehensive understanding of the underlying mechanisms of toxicity. We plan to expand this aspect in future studies to provide a more holistic view of dimethoate's effects.

We appreciate your constructive feedback, and we will revise the manuscript to better highlight the unique contributions of our work and outline potential areas for further exploration.

**Research group has used both male and female rats for the treatment. However, in results it has not shown comparison of responses from male and female rats. Results are analyzed in very narrow spectrum of treated Vs non-treated.**

**Response:** Thank you for your valuable feedback. We acknowledge the concern regarding the lack of a comparison between male and female rats in the results section of the manuscript. While both male and female rats were included in the study, the initial analysis focused primarily on comparing treated versus non-treated groups across all subjects, without emphasizing the gender-specific differences.

We agree that it is important to highlight any potential differences between male and female rats, especially given the possibility of gender-based variations in response to pesticide exposure. In light of your feedback, we have re-analyzed the data to specifically compare the hematological parameters between male and female rats in both treated and control groups.

We appreciate your suggestion to broaden the scope of our analysis, and we believe these revisions will strengthen the manuscript and provide more comprehensive insights into the effects of dimethoate exposure.

**There is lot of mismatches in description, table and figure**

**Response:** We sincerely thank you for pointing out the mismatches between the description in the manuscript, the tables, and the figures. We apologize for any confusion this may have caused and appreciate your careful review of the manuscript.

Upon re-examining the manuscript, we have identified and corrected the following discrepancies. We have carefully reviewed the tables and figures to ensure that they accurately reflect the descriptions and results presented in the text. All relevant values, statistical results, and trends now match the descriptions and are consistent across the manuscript. We have revised the figure legends and table titles for clarity, ensuring they are properly aligned with the data presented. We have also made sure that each figure and table is referred to in the correct order and that the legends provide accurate and complete explanations. We have ensured that all data points presented in the figures and tables are consistent with the results discussed in the manuscript. Any errors in the presentation of data have been corrected.

The manuscript has been updated accordingly, and we believe these revisions resolve the mismatches between the text, tables, and figures. We appreciate your attention to detail and trust that these revisions will improve the clarity and accuracy of the manuscript. Thank you for your understanding, and if further clarifications are needed, please do not hesitate to inform us.

**Reviewer 2:**

**The article looks interesting to me. I have a few minor comments that could be incorporated to improve it. 1. Since Figure 3.1 appears before Figure 2, please correct the figure numbering so that the figures appear in proper sequence.**

**Response:** Thank you for your positive feedback and valuable comments. We appreciate your careful review of the manuscript. Regarding your comment on the figure numbering, we have made the necessary corrections to ensure that the figures appear in the proper sequence.

We have updated the numbering of the figures so that Figure 3.1 now appears after Figure 2, and the rest of the figures have been renumbered accordingly to maintain proper order.

We believe this adjustment will improve the manuscript's clarity and organization. Thank you for bringing this to our attention, and please let us know if there are any other aspects that require further revision.

**In Figures 3.1, 2, and 3 (all bar diagrams), label the Y-axis clearly to indicate what is being measured.**

**Response:** Thank you for your helpful suggestion regarding the Y-axis labels in Figures 3.1, 2, and 3. We agree that clearly labeling the Y-axis is essential for improving the clarity of the figures.

In response to your comment, we have updated the Y-axis labels in Figures 2, 3, and 4 to clearly indicate the parameters being measured. The labels now explicitly describe the units of measurement and the specific variables being assessed, ensuring that the figures are more informative and easier to interpret.

We appreciate your attention to this detail and believe these revisions will improve the presentation of our data. Should you have any further suggestions, we would be happy to incorporate them.

**In the graphical abstract, please include the name of the dose for clarity.**

**Response:** Thank you for your thoughtful comment regarding the graphical abstract. We agree that including the name of the dose will enhance clarity and provide a more complete understanding of the study's methodology.

In response to your suggestion, we have updated the graphical abstract to include the name of the dose used in the study, along with the relevant concentration or dosage information. This addition ensures that the graphical abstract accurately reflects the key elements of the study and improves its overall clarity.

We appreciate your feedback and believe this update will make the graphical abstract more informative for readers. If you have any further suggestions, we would be happy to incorporate them.
